# A Retrospective Observational Study of Continuous Wireless Vital Sign Monitoring via a Medical Grade Wearable Device on Hospitalized Floor Patients

**DOI:** 10.3390/jcm13164747

**Published:** 2024-08-13

**Authors:** Grant B. Weller, James Mault, Maria Eloisa Ventura, Justin Adams, Frank J. Campbell, Kevin K. Tremper

**Affiliations:** 1 BioIntelliSense, Inc., Golden, CO 80401, USA; 2 Ardent Health Services, Nashville, TN 37027, USA; 3 Department of Anesthesiology, University of Michigan, Ann Arbor, MI 48109, USA; ktremper@med.umich.edu

**Keywords:** physiologic monitoring, vital signs, wearable electronic devices, clinical decision support systems, wireless technology

## Abstract

**Background:** Continuous vital sign monitoring via wearable technology, combined with algorithm-based notifications, has been utilized for early detection of patient deterioration. In this retrospective observational study, we summarize a large-scale implementation of a continuous monitoring system in medical–surgical units of two hospitals over the course of fifteen (15) months. **Methods**: An FDA-cleared wireless monitoring device (BioButton^®^, BioIntelliSense Inc., Golden, CO, USA), was placed on each patient upon admission. The wearable device measures heart rate and respiratory rate at rest, skin temperature, and patient activity levels. High-frequency data (up to 1440 measurements per day) are transmitted to display in exception management software (BioDashboard™, version 2.9, BioIntelliSense Inc.). Algorithmic and rules-based notifications are triggered based on clinical and statistical trending criteria. We present (i) agreement of device readings with bedside charted measurements, (ii) the frequency of notifications, (iii) the occurrence of notifications prior to clinical deterioration events, and (iv) impact on clinical management, including early data on length of stay (LOS). **Results**: In total, 11,977 patient encounters were monitored at two sites. Bias ±95% limits of agreement were 1.8 ± 12.5 for HR and 0.4 ± 8.0 for RR. The rates of notifications were 0.97 and 0.65 per patient-day at Sites 1 and 2, respectively. Among clinical deteriorations, 73% (66%) had at least one notification within 24 h prior at Site 1 (Site 2). At Site 1, there were 114 cases for which a notification led to a new or changed physician’s order. LOS in the first unit monitored by the system exhibited a decreasing trend from 3.07 days to 2.75 days over 12 months. **Conclusions**: Wearable continuous vital sign monitoring with the BioIntelliSense BioButton^®^ system enables early detection of clinical deterioration.

## 1. Introduction

Patients in intensive care units (ICUs) are closely monitored with both electronic devices and one-on-one highly trained nurses. Once patients are discharged to the floor, they receive intermittent, 4-to-8-hour vital sign checks and nurse-to-patient ratios of 1:4 or 1:5 for medical and surgical patients [1]. The traditional practices of disturbing patients for nighttime vitals have been questioned due to the adverse effects on healing due to disruption of sleep [2,3]. On the other hand, modified early warning scores (MEWSs), which are recommended to identify patients who are at risk of deterioration, require frequent assessment of vital signs [4,5]. For many patients, it may be best to skip nighttime vital signs and allow the bedside nurse to use their clinical judgement to decide who needs nighttime vitals [3]. Unfortunately, due to the variability of patients’ comorbidities, failure-to-rescue events continue to occur, leading to the consensus that floor patients need more frequent monitoring of vital signs [5]. It has also been documented that despite respiratory rate (RR) being an important predictor of deterioration, included in MEWSs and qSOFA, it is the most frequently missed and inaccurately recorded vital sign [6,7].

Recently, new wearable technologies have become available to passively monitor vital signs remotely and continuously. A comprehensive review of the literature evaluating the available devices concluded that to date the studies mostly involve validation and feasibility and are under-powered to assess clinical utility [4]. A recent retrospective, propensity-matched study found promising results utilizing remote monitoring on hospital floor patients [8].

The purpose of this report is to present our findings utilizing the FDA-cleared BioButton^®^, a coin-sized wireless continuous monitoring wearable device (BioButton^®^, BioIntelliSense, Inc., Golden, CO, USA) as part of standard care in a large clinical inpatient population. The primary objective of our analysis is to assess the agreement of BioButton-recorded vital sign readings (HR and RR) with concurrent manually charted bedside readings in the inpatient setting. The BioButton system is FDA-cleared (https://www.accessdata.fda.gov/cdrh_docs/pdf21/K212957.pdf, accessed on 10 July 2024) with a clinical accuracy specification of root mean square error (RMSE) of less than 5 beats per minute for resting heart rate in the range of 40–150 beats per minute and less than 3 respirations per minute for respiratory rate in the range of 5–35 respirations per minute.

Additionally, we assess the following aspects of the BioButton system as secondary objectives:

1. The frequency of physiologic notifications triggered by the data generated by the BioButton^®^ wearable device.
2. The correspondence and timing of BioButton notifications relative to the occurrence prior to clinical deterioration events (CDEs).
3. The impact of the BioIntelliSense system, BioButton wearable device, and centralized nurse monitoring on the clinical management of patients and length of stay (LOS) since the start of implementation.

## 2. Materials and Methods

Institutional Review Board exemption was obtained for this retrospective study. On 1 September 2022, we initiated a floor patient monitoring project at a rural community hospital in Texas (Site 1). The project was expanded to a second site, an urban community hospital in Oklahoma (Site 2) on 11 July 2023. The monitoring utilized the BioButton^®^ wearable medical device (BioButton^®^, BioIntelliSense, Inc., Golden, CO, USA). The initial implementation was conducted on one medical unit of 30 beds at Site 1 and expanded to additional units at Site 1 and, subsequently, Site 2 over time. As of 20 September 2023, BioButton monitoring had expanded to 119 non-ICU beds in 3 units at Site 1 and 250 non-ICU beds in 10 units at Site 2.

**Figure 1:**
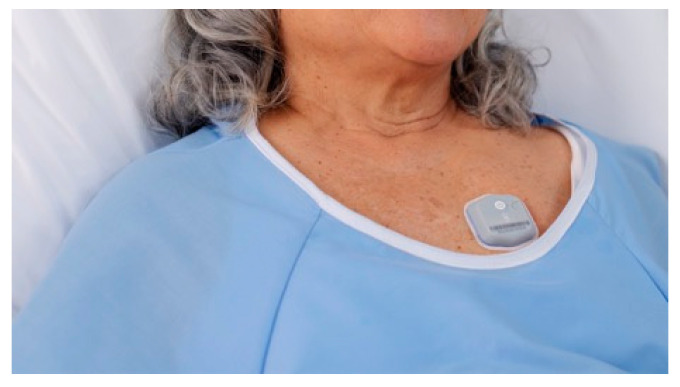
Placement of the BioButton monitoring device.

The BioButton wearable device was placed in the left subclavicular area, Figure 1, at the time of patient admission to the floor. The device transmits the following data: heart rate at rest (HR), respiratory rate at rest (RR), skin temperature (T), and patient activity level (low/medium/high) for up to seven days. HR, RR, and T values are captured once per minute, with HR and RR being reported only while the patient is at rest. The data are continuously transmitted via a Bluetooth gateway (the BioHub™, BioIntelliSense, Inc., Golden, CO, USA) which is configured and connected to the hospital Wi-Fi network. The data are processed on cloud-based software (BioCloud™, version 2, BioIntelliSense, Inc., Golden, CO, USA), which evaluates the data for clinical notifications in the clinical intelligence system (BioDashboard™, BioIntelliSense, Inc., Golden, CO, USA) and hourly median values are generated which are utilized for clinical documentation. Minute-by-minute data were not visible on the device or otherwise directly to staff at bedside. The RR and HR were securely transmitted to BioDashboard and the median values (for each hour of monitoring) were recorded in the electronic medical record (EMR, Epic systems, Verona, Wisconsin) within the vital sign flowsheet rows, alongside other sources of these same data types, without nursing validation.

Minute-by-minute data and notifications are displayed and reviewed through a clinician user interface (BioDashboard™, BioIntelliSense, Golden, CO, USA) that provides both census-level and individual patient-level views of the data. Clinical notifications based on the BioButton data were generated using BioCloud software and displayed to end users in the BioDashboard. Vital sign notifications were generated for the following conditions: high/low HR; high/low RR; double (simultaneous) high HR and RR; triple (simultaneous) high HR, RR, and T; and trending increases of at least 30% (over 2 h or 4 h) for HR/RR, Table 1. Notifications for high and low vital signs were based on hourly median values of these parameters.

Patients were monitored using the BioDashboard from a central nursing station, from 8:00 a.m. to 5:00 p.m., Monday through Friday, in general care areas of the hospital facility, allowing the surveillance nurse to notify and collaborate with the bedside nursing teams. At Site 1, a single dedicated nurse monitored multiple units (119 beds). An additional centralized nursing station at Site 2 monitored up to 250 beds. By applying both algorithmic and rules-based exception management, these medical and surgical patients were monitored by a single nurse. This centralized monitoring workflow was not previously used at either site and was enabled by the introduction of the BioIntelliSense technology.

When a notification triggers inside the clinical dashboard, it displays a red exclamation point icon, which indicates an active notification, and a red checkmark icon, which indicates a notification review is incomplete. The centralized monitoring nurse then reviews the notification, which typically includes a review of the patient chart in the EHR and a call or EHR-based message to the bedside provider. Previous reviews and documentation are saved so other staff can review the assessments. When the notification review is complete, the red checkmark disappears although the notification icon remains illuminated until the physiologic abnormality has resolved.

**Figure 2:**
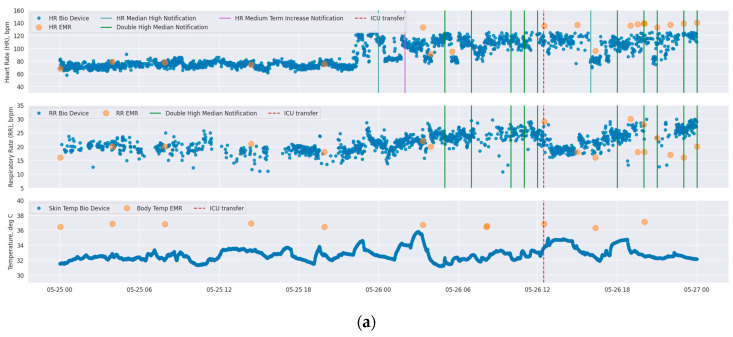

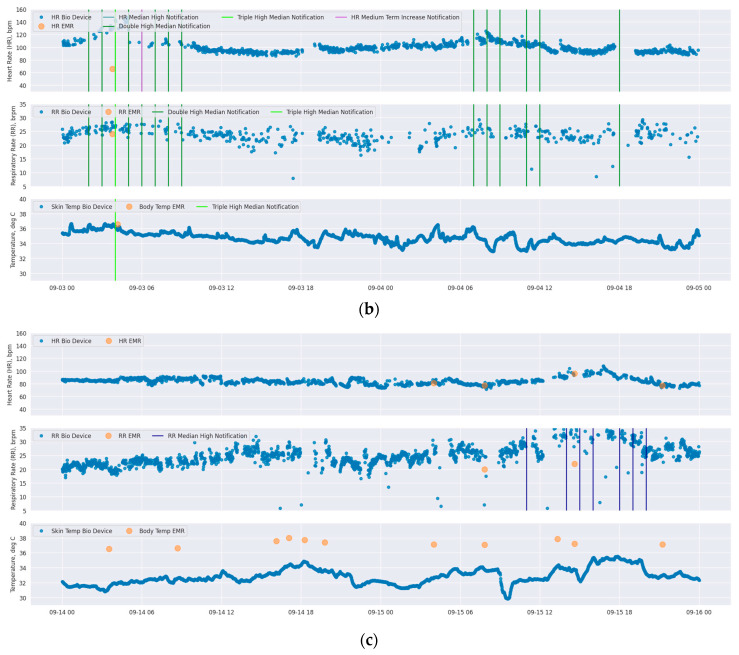
Time-series plots of vital signs of three different patients (each of subfigures (**a**–**c**) correspond to each individual patient) as measured by BioButton (blue points) and bedside charting (orange points). Each subfigure shows heart rate (**top**), respiratory rate (**middle**), and temperature (**bottom**; BioButton skin temperature and bedside body temperature). The timing of BioButton vital sign notifications for each of HR and RR are annotated on each plot (note that “double high” and “triple high” notifications are annotated on both HR and RR plots in dark green and light green, respectively). Date and local time are shown on x-axis.

To illustrate the potential of continuous monitoring in comparison with intermittent spot checking of vital signs, Figure 2 shows three time-series plots of BioButton-captured vital signs alongside spot-check readings obtained from the bedside. In the first case (2a), a new diagnosis of atrial fibrillation was found after the BioButton identified heart rate readings of over 120 bpm approximately five hours prior to the first bedside reading of tachycardia. In the second case (2b), tachycardia was detected by the BioButton despite being missed by a bedside check, and the patient was initiated on a beta blocking medication. In the third case (2c), the patient’s respiratory rates were recorded by the BioButton in the range of 25–35 respirations per minute despite a bedside reading of 22. The corresponding notifications for high respiratory rates led to follow-up testing for COVID-19 and influenza on this patient.

On 30 May 2023, the BioButton wearable device firmware was updated to accommodate an extended measurement range on both heart rate and respiratory rate at rest. Prior to this update, BioButton reported heart rates in the range of 40–125 beats per minute; this was expanded to 40–150 beats per minute. The respiratory rate at rest reporting range expanded from 10–30 to 5–35 respirations per minute. On 15 October 2023, a second, minor update to the device was implemented to increase vital sign accuracy.

Clinical deterioration events (CDEs) were defined by the incidence of a rapid response team (RRT) call, ICU transfer, or patient expiration. Events that occurred within 12 h of each other (e.g., an RRT call followed by an ICU transfer 2 h later) were analyzed as a single deterioration event. Manual chart review was conducted on these events to exclude RRT calls or ICU transfers that were unrelated to physiologic deterioration (e.g., ICU transfer for a planned procedure) as well as to exclude expiration of patients placed on hospice care. Clinical impact was assessed by identifying changes to physician orders documented by the central monitoring team at Site 1 and by examining hospital length of stay (LOS) of monitored patients over time.

The BioButton wearable device and BioIntelliSense System, along with supporting software tools, were implemented as part of standard patient care at both sites. De-identified data for this analysis were captured retrospectively from the BioIntelliSense BioCloud data system and the electronic health record (EHR) data of monitored patients.

Summary statistics describing patient encounters, vital sign readings, and notification rates were calculated for each site. Agreement between BioButton-measured HR/RR and corresponding bedside charted values recorded for the same minute was assessed via Bland–Altman limit of agreement analysis using data obtained since 15 October 2023, when the most recent update to device firmware was completed. All clinical deterioration (CD) events were reviewed to determine if, and when, notifications triggered in advance of the event. For notifications that preceded CD events the mean and median times of the first notification, and times of all notifications, were determined. In reporting on notification rates and clinical deterioration events, only data after 30 May 2023 were analyzed (the date of range extension updates to the wearable device firmware).

## 3. Results

**Figure 3:**
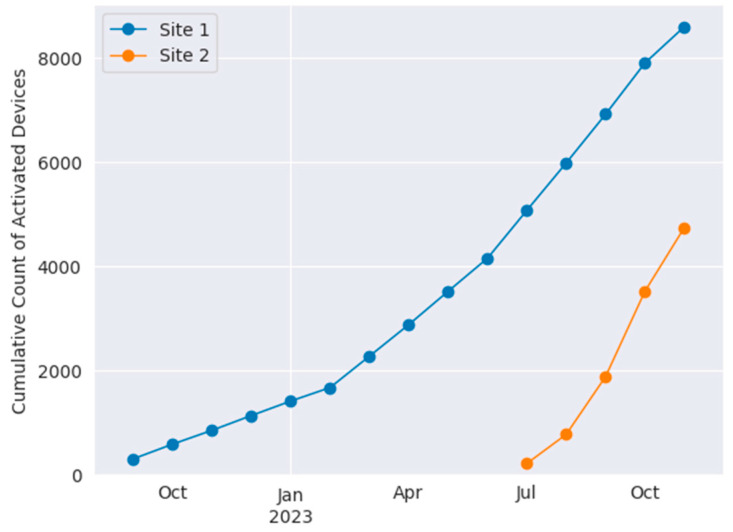
Cumulative use of BioButton wearable devices at Site 1 (blue) and Site 2 (orange).

A combined total of 11,977 patient encounters (7872 at Site 1 and 4105 at Site 2) were monitored at the two hospital sites from 1 September 2022 through 24 November 2023. The first unit at Site 1 was monitored for six months before expanding into additional units; at Site 2, monitoring was implemented in all medical–surgical units of the hospital within three months from installation of the first unit. A chart showing the growth of total activated wearable devices at each site is shown in Figure 3 below.

A total of 651,505 patient-hours of vital sign monitoring data was obtained. The mean (median) of monitored hours per admission was 69.6 (47.6) h at Site 1 and 78.2 (50.2) h at Site 2.

**Figure 4:**
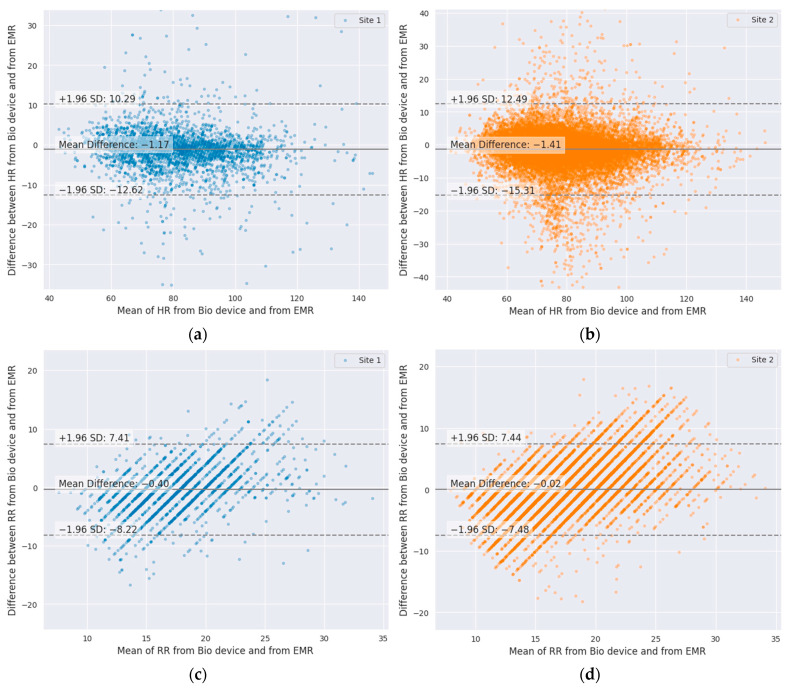
Bland–Altman plots of wearable-device-measured vital signs compared to same-minute charted readings: (**a**) HR, Site 1; (**b**) HR, Site 2; (**c**) RR, Site 1; (**d**) RR, Site 2.

Bland–Altman analyses of vital signs recorded by the BioButton wearable device matched to the same minute timestamp as a charted value are shown for HR and RR at each site in Figure 4. Analysis for Site 1 included 1449 simultaneous observations of HR and 1081 observations of RR from 479 admissions; at Site 2, there were 8113 HR pairs and 6172 RR pairs obtained from 1156 admissions. Mean differences (clinical bias) were within 2 beats per minute for heart rate and 0.5 respirations per minute for respiratory rate for both sites, Figure 4. Heart rate limits of agreement were similar between sites, with approximately 95% of ±12 (Site 1) and ±13 (Site 2). Respiratory rate analysis limits of agreement of ±7.5 were also similar at both sites.

An average of 0.97 and 0.65 vital sign notifications per patient per day were generated at Site 1 and Site 2, respectively. The frequency of the six types of notifications, for each site, is presented in Table 2. The most common notification was for high RR, with 0.24 and 0.23 notifications per patient per day at Site 1 and Site 2, respectively. Forty percent (4816) of patients did not trigger any notifications for their entire stay; this includes 39% of encounters at Site 1 and 43% at Site 2.

At Site 1, after the device firmware update on 30 May 2023, there were 124 CD events that occurred during BioButton monitoring; at Site 2, there were 71. Of the 124 patients who experienced CD events at Site 1, 90 (72.6%) were preceded by at least one notification within a 24 h period prior to the event; at Site 2, 47 (66.2%) were preceded by a notification.

Of all vital sign notification types, the high respiratory rate notification showed the highest sensitivity for deterioration events, with 37.1% (46 events with preceding notification) and 40.1% (28 events) at Site 1 and Site 2, respectively. This was followed by notifications for double high HR and RR, with 21.8% (27 events) and 19.7% (14 events), and high HR, with 21.0% (26 events) and 12.7% (9 events). Finally, low RR was notified in advance of 16.9% of events at each site (21 and 12 events, respectively). Examining false positive rates, high RR (11.3% and 10.7%) and low RR notifications (9.8% and 9.0%) were the highest among notification types. There were 16,318 monitored patient-days at Site 1 and 16,354 at Site 2 during which no subsequent patient deterioration was detected. The overall patient-day false positive rates (i.e., the proportion of patient-days without a deterioration event that also generated a notification) were 33.8% and 28.3% at Site 1 and Site 2, respectively.

**Figure 5:**
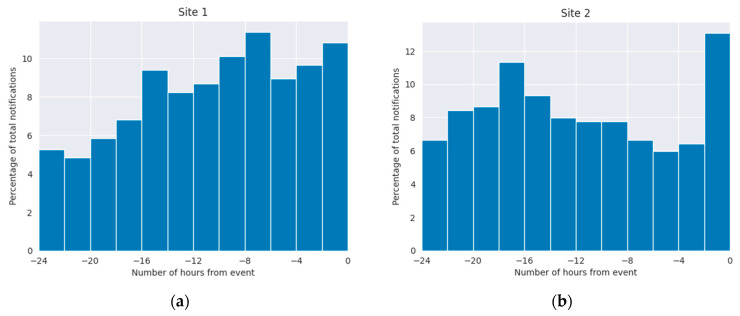
Timing of vital sign notifications relative to clinical deterioration events: (**a**) Site 1; (**b**) Site 2. Event time is aligned to time 0.

When notifications were generated in the 24 h period prior to incidence of deterioration events at Site 1, the first notification was triggered an average (median) of 14.8 (16.9) hours prior to the event. At Site 2, the first notification was generated 14.8 (16.1) hours prior to the event. Considering all notifications occurring in a 24 h period prior to deterioration events, notifications occurred most frequently in the period 4 to 18 h prior to the event, Figure 5. At Site 2, a spike in notification frequency in the two hours prior to event incidence is also observed.

Chart review of the 34 (27.4%) and 24 (33.8%), at Site 1 and Site 2, events that were not preceded by a BioIntelliSense BioButton system notification for HR or RR found that most were ICU transfers associated with a concern for low blood oxygenation (SpO2) or hypotension, occurring in the absence of significant observed derangement in resting heart rate or respiratory rate. Additional events were attributable to patient self-report of pain or concerning laboratory values.

While vital sign notifications occur in the absence of subsequent deterioration events, many of these notifications were in fact clinically useful and actionable. In addition to bedside nursing actions taken because of BioButton vital sign notifications, from May through November 2023, central monitoring staff at Site 1 documented 114 patient encounters during which there was a vital sign notification that ultimately led to a new or changed physician order or diagnosis. Among the new diagnoses were atrial fibrillation, sepsis, and a medication allergy. New or adjusted dosages of beta blocking medications were the most common medication-related orders resulting from BioIntelliSense notifications. Table 3 shows the monthly trends in length of stay (median and geometric mean, GMLOS) of patients monitored in each of the three units at Site 1 since the start of monitoring patients with the BioButton device. In the first unit to implement monitoring, GMLOS decreased from 3.07 days in October 2022 to 2.75 in October 2023. Similar decreasing trends were observed in two other units monitored at this site. Monitoring at Site 2 in this study covered fewer than four months, and it was too early to identify sustained trends in length of stay.

## 4. Discussion

It has been documented that respiratory rate is the most skipped and least accurately measured vital at night, despite RR being an essential component of determining a patient’s cardiopulmonary status [6]. There is also concern that once a patient leaves a highly monitored area, such as an ICU or post-anesthesia care unit (PACU), the assessment of the patient’s condition is reduced to every four hours or less. These assessments themselves can be extremely variable given the experience of the provider and the complexity and variability of the patients [7]. To assist the clinical providers on the patient floors scoring systems have been proposed to facilitate the early detection of patients whose condition may be deteriorating [5]. The modified early warning score (MEWS) is the most validated assessment tool [9]. But it requires accurate and timely determination of vital signs including RR. To resolve this dilemma of allowing patients much needed sleep, while at the same time having a higher frequency of vital signs assessment, it has been suggested that new wireless technologies be employed to provide this much needed surveillance to detect deteriorating patients [4].

A variety of wireless technologies have become available [4], and several of them have been studied for their performance in hospital settings. In all cases, real-world comparison of vital sign readings to bedside references shows deviation from results reported in highly controlled performance testing. Our findings comparing the BioButton vital sign readings to bedside measurements are similar to findings reported in a recent systematic review of inpatient studies [10], wherein the reported Bland–Altman limits of agreement (LoA) for heart rate measurement were (−8.8, 6.5) for HealthPatch (VitalConnect, San Jose, CA, USA), (−11.1, 10.7) for Visi Mobile (Sotera Digital Health, Carlsbad, CA, USA), and (−12.6, 9.5) for Visi Mobile in a separate study. For respiratory rate, reported Bland–Altman LoAs were (−15.8, 11.2) and (−10.3, 9.0) for HealthPatch and (−5.5, 7.9) for Visi Mobile.

For effective remote patient monitoring, the optimal device would be minimally intrusive to the patient while providing high-quality vitals at a frequency greater than once every 4 hrs and it would have a battery life longer than the patient’s average length of stay. In this study we present the results of our experience utilizing a small wireless wearable device which provides vital signs every hour and has a battery life of seven days for inpatient monitoring. In this retrospective review of a progressive rollout of this system there is no parallel control group, but its utility was assessed by noting the types and frequency of notifications which preceded CD events, modifications in care, and reductions in LOS. As might have been predicted, high RR was the most frequent notification. Since RR is a primary component of minute ventilation and arterial CO_2_, an increase in RR in a patient at rest may signify either a decrease in tidal volume associated with decreasing pulmonary compliance or an increase in metabolic rate associated with sepsis.

An increase in RR alone warrants a patient assessment for it has been associated with impending cardiopulmonary arrest [11]. Following this physiology, it is not surprising that the next most frequent notification is RR trending increase followed by a double high of RR and HR, Table 2.

Because of the difficulty of accurately measuring RR in floor patients, especially at night, it has therefore been referred to as the “neglected vital sign” [6]. Respiratory rate as manually charted has been consistently shown to exhibit bias and multiplication artifacts (e.g., counting breaths for 30 s and multiplying by two) [12]. It is therefore not surprising that our analysis of RR showed wide limits of agreement with bedside-measured RR, 95% of ±7.5. On the other hand, the fact that the RR (like HR) is measured continuously enables the possibility of using the device to reduce the frequency of interruptions for vital sign measurements in some patients.

This study presents some promising results with respect to detecting early signs of patient decompensation and allowing for intervention prior to more serious events. Additionally, direct evidence of patient impact was captured through documented changes to physician orders resulting from follow-up on vital sign notifications generated by the wearable device. Although as stated above, there was no control group for the assessment of improved safety or decrease in cost, there was, however, a trend in decreasing LOS which implies both improved care and decrease in cost. Additional studies are needed to demonstrate these promising improvements in outcomes through statistically controlled analyses.

There are limitations of this report in addition to issues associated with any observational study. First, there was no formal assessment of nursing staff acceptance and its possible effects on staff workload such as the potential effects of false positive notifications, impact on clinical workflow, and general provider satisfaction with the BioButton. Second, a significant limitation in this initial rollout was that the centralized nurse monitoring station was only staffed weekdays on the day shift. This decision was made for reasons of cost. One would expect a faster response to BioButton notifications if the monitoring station was staffed 24 h, 7 days per week. It is likely that more of the events with preceding notifications may have been acted upon earlier, especially over the weekends. Despite these limitations, the findings of this preliminary report are very promising in improving care by early detection of patient deterioration on medical and surgical floors. To our knowledge, this is the first report of such a medical grade wearable and system in this large a number of hospitalized patients.

## Figures and Tables

**Table 1:** BioButton vital sign notifications.

Notification Name	Description
HR Trending Increase	Notifies when the mean heart rate measured over the past 2 h exceeds the mean heart rate of the previous 4 h by more than 30% or the mean over the past 4 h exceeds the mean of the previous 24 h by more than 30%.
RR Trending Increase	Notifies when the mean heart rate measured over the past 2 h exceeds the mean heart rate of the previous 4 h by more than 30% or the mean over the past 4 h exceeds the mean of the previous 24 h by more than 30%.
Median HR Notification	Notifies when the median heart rate over the most recent 60 min of data is either >115 bpm (high notification) or <50 bpm (low notification).
Median RR Notification	Notifies when the median respiratory rate over the most recent 60 min of data is either >28 rpm (high notification) or <8 rpm (low notification).
Double High	Notifies when the median heart rate over the past 60 min is >100 bpm and the median respiratory rate over the past 60 min is >24 rpm.
Triple High	Notifies when the median heart rate over the past 60 min is >100 bpm and the median respiratory rate over the past 60 min is >24 rpm and the median skin temperature over the past 60 min is >36 °C.

**Table 2:** BioButton vital sign notification rates.

Notification Type	Notification Rate (per Patient per Day)	
	Site 1	Site 2
HR Trending Increase	0.06	0.06
RR Trending Increase	0.12	0.12
Median HR Notification	0.16	0.06
Median RR Notification	0.52	0.35
Double High	0.10	0.04
Triple High	0.02	0.01
Total	0.97	0.65

**Table 3:** Discharge count (N) and length of stay (median and geometric mean, GMLOS) of patients monitored with BioButton in Site 1 medical–surgical units, by month.

Month of Discharge	Unit 1			Unit 2			Unit 3		
	N	Median	GMLOS	N	Median	GMLOS	N	Median	GMLOS
Oct 2022	255	3.12	3.07	-	-	-	-	-	-
Nov 2022	241	3.01	3.23	-	-	-	-	-	-
Dec 2022	259	2.83	3.04	-	-	-	-	-	-
Jan 2023	241	3.15	3.25	-	-	-	-	-	-
Feb 2023	227	2.91	3.18	-	-	-	-	-	-
Mar 2023	242	3	3.09	242	3.11	2.91	-	-	-
Apr 2023	282	2.75	2.88	305	3.13	3.09	-	-	-
May 2023	219	2.61	2.91	257	3.3	3.12	-	-	-
June 2023	246	2.75	2.9	313	3.05	3.04	-	-	-
Jul 2023	250	2.57	2.72	311	2.83	2.83	309	2.95	2.94
Aug 2023	248	2.42	2.55	253	3.11	3.07	292	2.59	2.75
Sept 2023	227	2.83	2.79	304	2.78	2.76	312	2.38	2.48
Oct 2023	245	2.59	2.75	310	2.97	2.95	342	2.4	2.48

## Data Availability

The datasets presented in this article are not readily available due to data privacy restrictions.
